# Machine learning approaches for spatial omics data analysis in digital pathology: tools and applications in genitourinary oncology

**DOI:** 10.3389/fonc.2024.1465098

**Published:** 2024-11-29

**Authors:** Hojung Kim, Jina Kim, Su Yeon Yeon, Sungyong You

**Affiliations:** ^1^ Department of Urology, Cedars-Sinai Medical Center, Los Angeles, CA, United States; ^2^ Department of Pathology, University of Illinois at Chicago, Chicago, IL, United States; ^3^ Department of Computational Biomedicine, Cedars-Sinai Medical Center, Los Angeles, CA, United States

**Keywords:** machine learning, spatial omics, digital pathology, genitourinary, oncology

## Abstract

Recent advances in spatial omics technologies have enabled new approaches for analyzing tissue morphology, cell composition, and biomolecule expression patterns *in situ*. These advances are promoting the development of new computational tools and quantitative techniques in the emerging field of digital pathology. In this review, we survey current trends in the development of computational methods for spatially mapped omics data analysis using digitized histopathology slides and supplementary materials, with an emphasis on tools and applications relevant to genitourinary oncological research. The review contains three sections: 1) an overview of image processing approaches for histopathology slide analysis; 2) machine learning integration with spatially resolved omics data analysis; 3) a discussion of current limitations and future directions for integration of machine learning in the clinical decision-making process.

## 1 Introduction

Over the last decade, automation and digitization of laboratory processes have slowly transformed everyday practices in hospitals. Recent advances in computational pathology, especially with machine learning (ML) suggest an imminent revolution in the clinical decision-making process (1). There has been a steady build-up of resources to further support this transition. Adoption of digital pathology is becoming more common among medical centers, and large repositories such as The Cancer Genome Atlas (TCGA) have steadily been accruing digital tissue slides complemented by multi-*omics* profiles of each sample (https://www.cancer.gov/tcga). There have been ongoing research efforts to adopt ML and artificial intelligence (AI) for image analysis, with several tools already approved by the Food and Drug Administration (FDA) for use with radiologic images (https://www.fda.gov/). Utilizing ML for digital slide images analysis could help clinicians not only with diagnosis but also with risk stratification through predicting genetic alterations and classifying tumors based on meaningful features. Features such as microscopic morphology and expression pattern can be factored into the learning process, along with “sub-visual” level image features that may not be recognizable by human pathologists. This enables the development of more precise modeling of pathologies and therefore improved outcome prediction compared to traditional grading systems, contributing to precision medicine.

Spatially resolved omics is another field that ML may help clinicians overcome the limitations of traditional molecular testing methods. Analysis at a single-cell level has gained great popularity over the recent few years (2). Spatial multi-*omics* further augments single-cell technology by preserving the spatial information associated with each transcript or protein. The extracted expression profile is often combined with high-resolution hematoxylin and eosin (H&E) stained tissue slide images for complementary information on histological features (3). Computer vision can perform such cellular image analysis with minimal user intervention (4). Spatial profiling enables detection of unique features such as quantities of immune and stromal components that are associated with the tumor microenvironment (TME) in a spatial context (5). Employing such tools in research for identifying novel biomarkers will help clinicians identify with more ease in selecting patients who will benefit from targeted therapies, notably immunotherapy. Compared to the rapid gain of popularity in the development of ML algorithms, only a few tools are currently approved by the FDA or undergoing clinical trials.

In this review, we aim to provide the reader with an overview of currently available tools and methods in digital slide analysis and spatial multi-*omics*, with a focus on open-source tools. Selected studies are discussed to showcase the potential of ML in investigating urologic pathologies, to provide a reference for future research in the field of urology. Lastly, we discuss technical and practical problems that need to be addressed before clinical implementation.

## 2 Use of AI/ML in pathological diagnostics

### 2.1 Current trend of ML in digital pathology slide image analysis in urologic oncology

**Figure 1:**
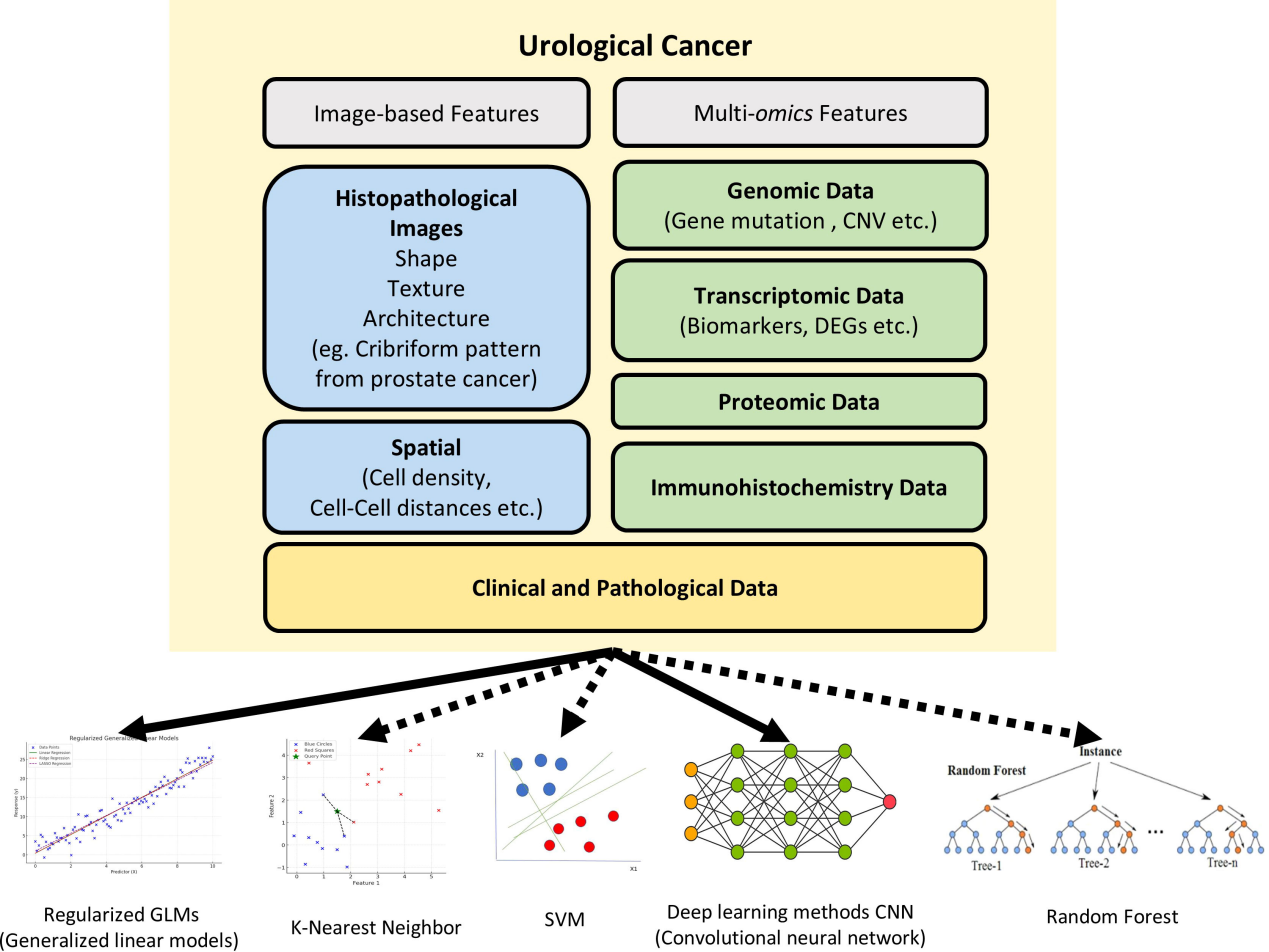
The various features and machine approaches used in urological cancer research. The recent urological cancer research used image features (blue), molecular features(green), and clinical features (yellow) for digital diagnosis. While solid lines indicate the machine learning models that are predominantly used in urological cancer research, dashed lines indicate machine learning models that have not yetbeen widely applied in this field. CNVs, Copy Number Variations; DEGs, Differentially Expressed Genes; SVM, Support Vector Machine.

There has been an influx of basic and translational research studies during the recent few years, mostly focused on developing new workflows powered by AI and ML methods. With rising availability of whole slide image (WSI) databases and rapidly improving performance of computational pathology, there have been efforts to detect and quantify novel features beside traditional histological features of nuclear and cellular morphology. Newer features often used for model development include immune cell infiltration, stromal composition, glandular architecture, and even topological features. Quantifying immune cell infiltration patterns can provide insights into the tumor microenvironment and potential response to immunotherapies. Analyzing stromal composition, such as the presence of cancer-associated fibroblasts or extracellular matrix components, can shed light on tumor invasiveness and metastatic potential. Additionally, assessing glandular architecture and its disruption can aid in grading and staging, especially for prostate cancer. Of surveyed studies, we identified urological specific features that had been used, including cribriform glandular pattern in prostate cancer and glomerular structure along with tubular morphology for kidney cancer (**Figure 1**). ML can aid in the discovery and quantification of such meaningful features. Furthermore, by integrating these features in model training, researchers are now moving beyond basic cancer detection, with the goal of developing more comprehensive and predictive models for tasks like survival prediction and treatment stratification. This represents a paradigm shift in the field, harnessing the power of ML and big data of pathology slide images to unlock new insights and improve patient outcomes.

Prostate cancer has garnered a relatively higher volume of research investigations compared to other malignancies of the urological system. This research emphasis can be attributed to the high incidence rates of prostate cancer and its substantial contribution to cancer-related morbidity and mortality. Huang et al. trained their model on randomly sampled tiles from H&E WSIs of The Cancer Genome Atlas (TCGA) Prostate Adenocarcinoma (PRAD) dataset. Cell-specific features including nuclear detail, glandular context and various TME elements including immune cells and stroma were extracted with a convolutional neural network (CNN) model to identify patterns predictive of early recurrence (6). In another study, a deep learning (DL) model was trained on the TCGA-PRAD dataset to predict TP53 mutation status from WSIs (7).

Similar efforts have also been made for bladder and renal carcinomas. Jiang et al. extracted TME features from TCGA bladder urothelial carcinoma (BCLA) WSI samples using CNN, which were then used to cluster the images by the K-means method. The clusters were found to correlate with prognosis and immune scores, suggesting there are differences in reactivity to immune checkpoint inhibitors. The authors furthermore attempted to quantify the TMI characteristics through an AI score, where a higher AI score predicted a higher therapeutic response to immunotherapy (8). Studies investigating renal cell carcinoma are relatively fewer in number compared to those focused on prostate and bladder cancers. Chen et al. developed a ML-based pathomics signature for clear cell renal cell carcinoma, where 5 most prognostic image factors were selected through least absolute shrinkage and selection operator (LASSO) analysis and factored into the ML formula (9).

### 2.2 Open-source tools for digital pathology analysis

While numerous studies have explored leveraging ML for digital pathology image analysis, many models developed by individual researchers and the complete methods and codes remain inaccessible to the public domain. This could perhaps be attributed to potential plans for patenting or commercializing the developed workflows. Future researchers could take advantage of several open-source tools, although optimization for individual research purposes might be necessary. **Table 1** shows a summary of the most popular open-source tools and programs currently available. Those tools are freely available to the public, and a wide range of extensions or plugins could be used in addition to further expand the utility of the main tool. For instance, Stardist can complement QuPath by using a pre-trained DL algorithm for nucleus detection. As such, open-source tools offer great flexibility in the functionality and the type of stains and data they can analyze.

**Table 1:** Open-source tools available for digital pathology image analysis.

Programming language	Tool	Functions	Stains	WSI	ML methods	Examples of application
Java	QuPath	project management, preprocessing, annotation, segmentation, classification, object detection, density mapping, biomarker quantification	Brightfield, fluorescence stains	WSI	RF	(10)
Java	ImageJ/Fiji	preprocessing, annotation, segmentation, classification, object detection, quantification, etc. depending on the plugins used	Brightfield, fluorescence stains	WSI	With plugins	(11)
Java	TMarker	segmentation, classification, nuclei counting, IHC staining estimation, regression	IHC	TMA	RF, SVM	(12)
Java	Orbit	annotation, segmentation, classification, object detection, quantification	Brightfield, fluorescence stains	WSI	RF, SVM	(13)
Java	Cytomine	project management, annotation, segmentation, classification, object detection, quantification	Brightfield, fluorescence stains	WSI	CNN	(14)
Python	Digital Slide Archive/HitomicsTK	TCGA data searching and downloading, project management, annotation, analysis through integration with other tools	Brightfield stains	WSI	–	(15)
Python	CellProfiler	preprocessing, annotation, segmentation, classification, object detection, quantification	Brightfield, fluorescence stains	Sections	U-net, CNN	(11)
Python	Ilastik	preprocessing, annotation, segmentation, classification, object detection, quantification, density mapping	Brightfield, fluorescence stains	Sections	RF	(16)
Java	ICY	project management, annotation, analysis through integration with other tools	Brightfield, fluorescence stains	WSI	With plugins	(14)
Python	PathML	preprocessing, segmentation, classification, analysis through integration with other tools	Brightfield, fluorescence stains	WSI	CNN	(17)

RF, Random Forest; SVM, Support Vector Machine; CNN, Convolutional Neural Network; U-net, An Encoder-Decoder Convolutional Neural Network; IHC, immunohistochemistry; TMA, Tissue Microarray.

QuPath utilizes deep learning methods like StarDist, which uses CNN trained on annotated image data to precisely segment and identify individual nuclei, for 2D and 3D nucleus detection (2). ImageJ, with its Trainable Weka Segmentation plugin (Fiji plugin), uses pixel classification approach for image segmentation. Pixel classification strategy means that each pixel in the image is transformed into a feature vector capturing properties such as intensity values, edge information, texture, etc. If the user manually annotates a subset of pixels, labeling them into distinct classes like cells or background, these labeled pixels serve as training examples for a ML classifier, such as a random forest (RF) or support vector machine (SVM). Once trained, the classifier predicts class labels for all remaining unlabeled pixels in the image. This tool often employs supervised learning methods where users annotate training data to teach the model (18). TMarker employs ML algorithms such as random decision trees and SVM to improve tumor and cell segmentation. Superpixels, which is an algorithm starting with a rough initial division of pixels and updating the clustering until the result meets a certain criterion, are used to segment the tissue image, and these segments are classified into foreground and background, and subsequently into malignant and benign categories (19). Orbit uses object classification through a linear SVM to differentiate objects within images. The trained SVM model can then predict the class label for all segmented objects in an image based on their feature representations (20). CYTOMINE is an open-source web platform enabling collaborative analysis of multi-gigapixel biomedifcal images through an integration of manual annotation tools and ML algorithms. It allows multiple remote users to access and create semantic annotations on shared images by labeling regions of interest with ontology terms, providing ground truth data for training models (21). The Digital Slide Archive (DSA) is a comprehensive platform designed to handle large imaging data sets by offering capabilities for storage, management, visualization, and annotation. The DSA is composed of an analysis toolkit enabling users to perform a variety of image analysis tasks (HistomicsTK), an interface that allows users to visualize slides and label annotations (HistomicsUI), a database layer for storing and managing the vast amounts of image data and associated metadata (MongoDB), and a web-server providing API for interacting with the platform and managing data (Girder) (22). CellProfiler, which is open-source software developed by the Broad Institute, employs supervised learning to classify objects based on their properties. CellProfiler first measures various features of each cell/object. These quantitative measurements extracted by CellProfiler pipelines serve as the feature vectors input to the ML classifier. Once trained on the user-provided labeled examples, the classifier model can automatically score and classify all objects in the dataset based on their measured features. It is compatible with various data analysis tools and supported by a robust user community and comprehensive documentation, including tutorials and forums (23). Ilastik utilizes random forests for pixel classification and segmentation. ICY is an open community platform for both applied mathematicians developing new algorithms and biologists seeking a powerful and intuitive tool for image analysis (24). Lastly, PathML applies DL models to automate and enhance histopathology image analysis, making these tools pivotal in advancing bioimage informatics through ML. PathML integrates with PyTorch and TensorFlow, which is a DL framework for model development, training, and inference using CNN, to enable training and evaluating DL models on standardized pathology datasets (25).

However, although there are many tools based on various ML models, almost all urologic cancer research has primarily utilized open-source tools based on CNN, with other ML models not being extensively applied yet (**Figure 1**). Wen et al. evaluated the performance of SVM, RF, and CNN for nucleus segmentation in breast and pancreatic cancer. The area under curve (AUC) values for breast cancer was 0.54 for SVM; 0.67 for RF; and 0.82, 0.84, and 0.86 for 3 runs of CNN. Similar results were seen with pancreatic cancer with AUC 0.47 for SVM; 0.42 for RF; and 0.69, 0.79, and 0.80 for CNN. Three runs were performed with CNN as the randomness in the data augmentation process presents different classification results across runs. Although CNN outperformed the two other algorithms in accuracy, its processing time was significantly longer than that of SVM and RF, with RF being the fastest method (26). Another study on cervical intraepithelial lesions and malignancy used CNN for feature extraction then compared KNN, RF, and SVM classification models on the extracted features. The stacked classification models were found to have higher accuracy compared to purely CNN classification, with the accuracy of the CNN model being 70.83%, and the accuracy of the CNN-KNN, CNN-RF, CNN-SVM models being 85.83%, 80.83% and 86.67%, respectively. This suggests stacking of different ML methods may be able to achieve better performance than a single classifier (27).

QuPath’s pixel classifier has been used on immunohistochemistry (IHC) stained images of prostate cancer for quantification of TME to predict response to immune checkpoint inhibitors (10) (18). In muscle invasive bladder cancer, tumor-stroma ratio (TSR) quantified by ML was linked to prognosis (28). The cell classifier for TSR calculation was based on a previously developed ML algorithm (29) and QuPath. Tools other than QuPath have also been utilized in other studies, although less often in urology. CellProfiler was used for feature extraction from H&E images for diagnosis of clear cell renal cell carcinoma (30). On the more practical side, QuPath was used to train ML algorithms for automated Ki-67 index quantification in prostate cancer tissue microarray (TMA) samples to assess PSA recurrence risk (31). This could possibly streamline Ki-67 assessment, which is an important prognostic indicator along with Gleason grade in prostate cancer.

There are several AI tools that have been validated with the large clinical trial cohorts and approved by the FDA that are clinically available. Paige Prostate (https://paige.ai/diagnostic-ai/) is so far the only AI tool in urologic pathology approved for whole slide image analysis (32). It uses scanned slide images to detect possible areas of prostate cancer based on morphology and p63 staining pattern. Artera AI (https://artera.ai) is another AI tool that predicts risk of progression and predicts the treatment response based on the histomorphology and patient’s clinical data (33). A few clinical trials are underway for evaluation and implementation of newly developed tools. Ramon et al. developed a deep learning algorithm for predicting FGFR alterations from H&E WSIs of bladder cancer and pan-tumor datasets including prostate cancer (34). This could help lessen the tumor screening burden in determining eligibility for treatment with erdafitinib, thereby improving access to targeted therapy. Another clinical trial aims to evaluate an AI algorithm for predicting response to adjuvant BCG (Bacillus Calmette-Guerin) treatment in non-muscle invasive bladder cancer (35). However, it should be noted that the AI tools are not recommended for autonomous diagnosis but as an assistance tool for the pathologists.

## 3 Machine learning in guided spatial profiling

There are several well-established commercial platforms available for spatial transcriptomics (ST) analysis. Notable platforms that use image-based ST technologies include Xenium of 10X Genomics, which improves on CARTANA by combining *in-situ* sequencing (ISS) with *in-situ* hybridization (ISH). MERSCOPE and CosMx Spatial Molecular Imager of NanoString are both based on ISH. Sequencing-based technologies are used in several array-based ST platforms such as Visium of 10X Genomics, BMKMANU S1000 of Biomarker, and Slide-seq. Stereo-seq achieves an even lower resolution and is available as STOmics by BGI. GeoMx Digital Spatial Profiler implements microdissection technology instead of microarray (36).

While spatial proteomics still lags behind ST, a few tools and technologies are available for use. CO-Detection by indEXing (CODEX; PhenoCycler™) allows highly multiplexed protein detection through sequential rounds of antibody staining and imaging, visualizing up to 60 proteins in a single section (37). Hyperion Imaging System combines mass cytometry with imaging for high-dimensional protein analysis (38). Multiplexed Ion Beam Imaging (MIBI) employs secondary ion mass spectrometry with metal-tagged antibodies for multiplexed detection and spatial resolution at the cell level (39). CyteFinder combines high-content imaging with multi-parameter protein detection and allows for the spatial localization and quantification of proteins in tissue sections. The IMS (Imaging Mass Spectrometry) platforms from Bruker uses Matrix-Assisted Laser Desorption/Ionization (MALDI) mass spectrometry (MS) imaging to map protein distribution within tissue sections at high spatial resolution and represents a cutting-edge approach in spatial proteomics (40). This platform utilizes MALDI for sample ionization and Fourier Transform Ion Cyclotron Resonance (FTICR) for mass analysis, enabling the detection and spatial localization of a broad range of biomolecules with high mass accuracy and resolution. The GeoMx DSP is also used to quantify protein expression in spatially resolved sections (41). These platforms are crucial for providing invaluable data to advance our understanding of the spatial and functional roles of RNAs and proteins in tissues and provide insights into cellular processes and disease mechanisms.

ML methods are increasingly being incorporated into spatially resolved transcriptomics and proteomics analysis. Deep learning models including CNN are often used for automatic feature selection and pattern recognition in imaging-based spatial omics, implemented as some of the open-source tools for digital pathology discussed above. There are several image analysis tools developed specifically for spatial omics analysis; Squidpy and Giotto utilize various ML-based methods for image analysis and visualization (42, 43). Spatial profiling offers measurement of several unique features that cannot be measured with simple single cell analysis, such as cell density, cell to cell interaction, cell proximity, and aggregation. Various ML methods and closely related statistical models are used to contextualize these measurements and carry out different tasks at hand (44, 45). Common tasks in spatially resolved transcriptomics analysis include spatial clustering, spatially variable gene (SVG) detection, cell type deconvolution, and identification of cellular interactions.

SpaCell and StLearn extract histological features from slide images via ResNet50, a convolutional neural network model pre-trained on ImageNet, then integrates the information with gene expression data in clustering (46, 47). SpaGCN is based on a graph convolutional network that works by extracting RGB values of each pixel which then separates spots into different spatial domains by unsupervised iterative clustering. SVGs (Spatially Variable Genes) can then be identified for each spatial domain (48). Supervised learning approaches can also aid in spatial cell type deconvolution, where traditional scRNA-seq deconvolution methods may not work as well. Robust Cell Type Decomposition (RCTD) is a supervised learning approach that leverages maximum likelihood estimation using annotated single-cell RNA-seq (scRNA-seq) data to infer cell types of proportions for each pixel (49). Cell2location uses a hierarchical Bayesian model to estimate the absolute abundance of cell types at a location based on predefined cell type signature sets (50). Spatial-ID employs a deep neural network (DNN) model pretrained on scRNA-seq datasets to produce cell type probabilities distributions (51). To infer intercellular signaling, MISTy trains a RF model for each target feature to derive feature importance scores. NicheNet observes the underlying spatial interactions and networks through a graph affinity algorithm based on predefined ligand-receptor pairs (52).

Spatial proteomics have also benefited from the integration of various ML methods into the data analysis pipeline (53). Much like other MS-based proteomics data, MS-based spatial proteomic data often suffers from missing measurement values. K-nearest neighbors (KNN) imputation is often used for missing value imputations before downstream analysis. Various ML classifiers may be used for protein localization prediction, including SVM, RF, KNN, neural networks, and others as mentioned in a review (54). pRolocs is an R package based on a SVM classifier for protein localization (55). MetaMass performs K-means clustering and assigns each cluster a location based on its marker content, available for Excel and R (56). TRANSPIRE uses a probabilistic Gaussian process classifier trained on organelle protein markers to predict protein translocation (57) (**Table 2**).

**Table 2:** Overview of machine learning applications in guided spatial profiling.

Category	Tools	Functions
ML for Spatial Transcriptomics	Squidpy, Giotto	ML methods for analyzing and visualizing spatial data, extracting features like cell density, cell proximity, and cell to cell interactions.
ML for Spatial Clustering	SpaCell, StLearn	Use deep learning to extract spatial features from histological images and cluster spatial domains, integrating gene expression data.
SVGs (Spatially Variable Genes) Detection	SpaGCN	Cluster spatial spots using RGB values and identify SVGs (Spatially Variable Genes) within each domain.
Cell Type Deconvolution	RCTD, Cell2location, Spatial-ID	Supervised ML models to estimate cell type probabilities distributions using spatial transcriptomics data.
Intercellular Signaling & Cellular Interactions	MISTy, NicheNet	Predict cellular interactions by analyzing spatial relationships and ligand-receptor pairs.
ML in Spatial Proteomics	pRolocs (R package), MetaMass, TRANSPIRE	ML models for protein localization prediction, aiding spatial proteomic analysis.

While there is a relative abundance of basic research focused on biomarker discovery and understanding molecular mechanisms, there is a need for more translational research that bridges the gap between basic and clinical studies by testing new techniques and methods on animal and patient-derived xenograft (PDX) models. Zimmerman et al. designed multiplex ISH probes that target the protein coding genes of the mouse and human transcriptome to integrate the transcriptome with histological features in diabetic kidney disease (DKD) (58). The development of such whole transcriptome panels across multiple organisms enables further discoveries through translational and clinical studies. Wang et al. modeled a spatially resolved metabolic network of the prostate cancer TME to predict selective metabolic targets for cancer cells (59). Although the application of spatial omics technology in the clinic is still in its development stage, image analysis combined with spatial omics technology offers great potential in improving clinical practices. Well-trained deep learning algorithms could extract meaningful spatial features and molecular patterns from spatial datasets, identifying new biomarkers and aiding clinical decision making.

## 4 Challenges and future directions

### 4.1 Common challenges in adopting ML into clinical practice

The integration of AI and ML in medicine faces significant real-world challenges, particularly in quality assurance and regulatory compliance. Like other medical devices, AI/ML tools are regulated by the FDA, and developing these regulations is a meticulous process. The College of American Pathologists (CAP) Advocacy Committee is actively working with government agencies to ensure fair and effective regulation of AI in pathology.

Real-world performance and quality assurance also present notable technical hurdles. Similar to traditional diagnostic tests, AI tools must undergo rigorous validation and regulatory scrutiny, as incorrect results can have serious implications for patient care. Despite the promising potential of ML, there are currently limited published examples in urologic cancers where they were tested and validated in a clinical trial setting. Key challenges on the research perspective include limited robustness, reproducibility, comparability, and interpretability. Current ML research heavily relies on retrospective data analysis, often notably using the TCGA datasets. This raises concerns about dataset biases and the generalizability of developed models. Prospective multicenter validation with adequate sample sizes is crucial to assess the robustness of ML algorithms before clinical implementation.

Furthermore, the relative lack of standardized data formats, and consistent outcome measures across studies makes it challenging to reproduce and objectively compare existing ML models. Publishing publicly available datasets, standardizing data collection protocols, and sharing developed algorithms as open-source resources could improve reproducibility and facilitate benchmarking.

Integrating ML into clinical workflows presents additional financial and operational challenges that must be considered. Many models suffer from the “black box” effect, where the decision-making process is opaque and difficult to interpret for clinicians. Improving transparency and providing clinically relevant outputs, such as diagnostic reports, could enhance trust and adoption of ML tools by clinicians. The cost for adoption of ML-based workflows should also be taken into account. Storing and processing large volumes of high-dimensional whole slide image data imposes significant computational demands, necessitating investments in hardware and computing resources. Continuous monitoring and updating of ML models incur additional recurring costs. Dedicated resources should also be set aside for integrating ML models with existing laboratory information systems. The final barriers to clinical implementation may be regulatory and ethical requirements and lack of clear guidelines for approving ML-based decision support tools. Although a clear guideline is yet to be established, addressing ethical concerns surrounding data privacy and liability is essential for acceptance among clinicians and patients. Overcoming these multifaceted challenges requires substantial financial investments and interdisciplinary collaborations.

### 4.2 Limitations for clinical application of spatial and single cell technologies

In current studies, there are efforts to apply single cell genomics to diagnostics. One study utilized single-cell genomic analysis to assess prostate cancer risk from prostate biopsy samples. Researchers employed single nucleus sequencing (SNS) to help the diagnosis. The sequencing examined copy number variations in individual cells, developing methods to identify clonal cell populations and reconstruct phylogenetic relationships among them. The genomic data was integrated with histopathology and anatomical information using a custom visualization tool named the Single Cell Genome Viewer (SCGV). The author concluded that SNS has the potential to enhance prostate cancer diagnosis and risk assessment from biopsies by providing more detailed genomic information than standard histopathology alone (60).

Technical limitations to clinical application of ST include complexity and repeatability. The complexity of the data and the need for sophisticated computational tools can be a barrier in clinical settings. Many spatial and single-cell omics methods require extensive sample preparation and processing, which can be time-consuming and not scalable for high-throughput methods. This limits their practicality for routine clinical use where quick turnaround time is often critical. The equipment for spatial and single-cell analyses is often expensive, making it difficult to implement these technologies widely in clinical settings, particularly in resource-limited environments. These complex and time-consuming procedures involved in preparing and analyzing samples using spatial omics technologies limit their feasibility for routine clinical use. Integrating and interpreting data from spatial and single-cell analyses with traditional clinical data (e.g., histopathological assessments, clinical imaging) may also prove to be challenging. Additionally, one of the primary challenges is that the profile obtained may not be representative of the entire tumor. The spatial heterogeneity within tumors means that sampling a small fraction of the tumor might not capture all relevant biological phenomena, leading to incomplete or skewed data. As for spatial proteomics, it should be noted that it is currently challenging to measure the whole proteome due to technological limitations. Advances are needed in the development of more specific antibodies and techniques beyond mass spectrometry-based methods to enhance the range and accuracy of protein detection. Although spatial technology shows more potential as a research tool than a clinical diagnostic tool due to these challenges, addressing these limitations could significantly enhance its clinical applicability.

### 4.3 Underrepresented urological malignancies

There exists a bias in the types of urological carcinomas being investigated, with a relative abundance of studies focusing on prostate cancer compared to kidney and bladder cancers. Additionally, several other urological malignancies remain understudied due to their lower incidence rates, such as testicular cancer, upper tract urothelial carcinoma, and penile cancer. Such underrepresentation of these cancers in research efforts underscores the necessity of addressing this disparity.
